# p53 regulates mtDNA copy number and mitocheckpoint pathway

**DOI:** 10.4103/1477-3163.50893

**Published:** 2009-05-06

**Authors:** Mariola Kulawiec, Vanniarajan Ayyasamy, Keshav K. Singh

**Affiliations:** Department of Cancer Genetics, Roswell Park Cancer Institute, Elm and Carlton Streets, Buffalo, NY, 14263, USA

**Keywords:** Cell cycle, metabolic stress, mitocheckpoint, mitochondrial, mitochondria, mtDNA, p53

## Abstract

**Background:**

We previously hypothesized a role for mitochondria damage checkpoint (mito-checkpoint) in maintaining the mitochondrial integrity of cells. Consistent with this hypothesis, defects in mitochondria have been demonstrated to cause genetic and epigenetic changes in the nuclear DNA, resistance to cell-death and tumorigenesis. In this paper, we describe that defects in mitochondria arising from the inhibition of mitochondrial oxidative phosphorylation (mtOXPHOS) induce cell cycle arrest, a response similar to the DNA damage checkpoint response.

**Materials and Methods:**

Primary mouse embryonic fibroblasts obtained from p53 wild-type and p53-deficient mouse embryos (p53 -/-) were treated with inhibitors of electron transport chain and cell cycle analysis, ROS production, mitochondrial content analysis and immunoblotting was performed. The expression of p53R2 was also measured by real time quantitative PCR.

**Results:**

We determined that, while p53 +/+ cells arrest in the cell cycle, p53 -/- cells continued to divide after exposure to mitochondrial inhibitors, showing that p53 plays an important role in the S-phase delay in the cell cycle. p53 is translocated to mitochondria after mtOXPHOS inhibition. Our study also revealed that p53-dependent induction of reactive oxygen species acts as a major signal triggering a mito-checkpoint response. Furthermore our study revealed that loss of p53 results in down regulation of p53R2 that contributes to depletion of mtDNA in primary MEF cells.

**Conclusions:**

Our study suggests that p53 1) functions as mito-checkpoint protein and 2) regulates mtDNA copy number and mitochondrial biogenesis. We describe a conceptual organization of the mito-checkpoint pathway in which identified roles of p53 in mitochondria are incorporated.

## INTRODUCTION

During cell cycle progression, major transitions between different phases are monitored by checkpoints that signal pathways. Checkpoints protect dividing cells from the consequences of genotoxic or nongenotoxic stress.[1,2] The cell cycle is delayed in response to exogenous stress signals, the shortage of available nutrients or in order to repair DNA damage.[3,4] Defects in the cell cycle checkpoint lead to the development of chromosomal rearrangements and promote tumorigenesis.[5]

Mitochondria perform multiple essential functions. These functions include, but are not limited to, controlling programmed cell-death,[6,7] and chromosomal rearrangements and epigenetic changes in the nucleus.[8,9] The existence of a mitochondria-checkpoint (referred to as the mito-checkpoint) signaling pathway(s) has been hypothesized, that maintains mitochondrial integrity and protects cells from the cellular consequences of damaged or dysfunctional mitochondria. The mito-checkpoint involved in maintaining the functional state of mitochondria may either delay cell-proliferation or induce cell-death, depending on the severity of the mitochondrial damage. In support of the existence of a mito-checkpoint in human cells, it has been demonstrated that the depletion of mtDNA results in cell cycle arrest in a variety of cell types.[10,11] Studies also show that decreases in ATP levels cause cell cycle arrest.[12–15] The defects in mitochondria have also been shown to trigger genetic[11,16] and epigenetic[9] responses. Notably, yeast *Saccharomyces cerevisiae* contains an elaborate and sophisticated regulatory pathway(s) that monitor(s) and respond(s) to defects in mitochondria. This pathway in yeast is controlled by retrograde regulatory genes RTG1, 2 and 3.[17–19] These genes in yeast appear to function as mito-checkpoint genes.[20] This argument is further supported by studies involving yeast cell division cycle (cdc) mutants. Interestingly, cdc28 and cdc35 show decreased mitochondrial biogenesis[21] and cdc5 and cdc27 show defects in mitochondrial segregation[22] as well as in nuclear division. Other examples include cdc8 and cdc21 mutants defective in nuclear DNA replication during the S phase of the cell cycle. The products of cdc8 and cdc21 are required for both nuclear and mitochondrial DNA replication.[23]

It has been suggested that p53 regulates mitochondrial oxidative phosphorylation (mtOXPHOS).[24] Indeed p53 plays a key role in many cellular processes, including apoptosis, genomic stability and tumorigenesis.[25,26] p53 also functions as a checkpoint protein after DNA damage.[27] In this paper, we report that p53 functions as a checkpoint protein after damage to mitochondria by mtOXPHOS inhibitors.

## MATERIALS AND METHODS

### Cell-lines and Culture Conditions

Primary Mouse Embryonic Fibroblasts (primary MEFs) obtained from p53 wild-type mouse embryos (p53+/+) and p53-deficient mouse embryos (p53 -/-) (kindly provided by Dr. S. Jones, University of Massachusetts Medical School, Worcester, MA) were cultured in DMEM medium supplemented with 10% (v/v) FBS, 100 *μ*g/ml streptomycin and 100 U/ml penicillin. Human colon carcinoma cell-lines HCT116 with wild-type p53 (p53+/+) and disrupted p53 (p53 -/-), kindly provided by Dr. Bert Vogelstein,[28] were cultured in McCoy's 5A medium supplemented with 10% (v/v) fetal bovine serum (FBS), 100 *μ*g/ml streptomycin and 100 U/ml penicillin. The cells were incubated in 5% CO_2_ at 37°C, then split when they reached 70% confluency. All experiments were performed at a cell density of 5 × 10^5^ cells/ml.

### Chemicals and Antibodies

Antimycin, oligomycin, rotenone, potassium cyanide and CCCP were purchased from Sigma (Sigma, St. Louis, MO). TTFA was purchased from Calbiochem (Gibbstown, NJ). All drugs were stored as stock solution at −20°C: oligomycin in DMSO; antimycin, rotenone, CCCP and TTFA in ethanol. Potassium cyanide in water was freshly prepared prior to treatment. The final concentration of drug vehicles in the cultures was <0.1%, so that it would not affect the viability or growth of the cell lines. Immediately before the experiment, the drugs were diluted in water and added to the culture dishes, giving a final concentration of 2.5–10 *μ*M.

### Cell Cycle Analysis by PI Staining

Approximately 10^6^ cells were detached by trypsinization at each time-point, washed twice in ice-cold PBS and fixed by adding, in drops, 2 ml of 70% ice-cold ethanol while vortexing and then placing them, at least overnight, at 4°C. Prior to flow cytometry, the analysis cells were pelleted out of the ethanol and then washed once with ice-cold PBS and once at 0.5% BSA in PBS and re-suspended in 1 ml of propodium iodide staining solution.[29] The cells were stained for more than 1 h by incubation at 4°C. Samples were analyzed for DNA content by FACSscan (Becton-Dickinson, Mountain View, CA). Ten thousand events were analyzed for each sample. After data acquisition, CellQuest software (Becton Dickinson, Franklin Lakes, NJ) and ModFit 5.2 software (Verity Software House, Topsham, ME) were used to analyze nuclear DNA distribution.

### Measurement of ROS production

Analysis of ROS production was done as described in Desouki *et al*.[30] Measurement of total intracellular ROS production was done using peroxide sensitive fluorescent probe CM-H_2_ DCFDA (Invitrogen, Molecular Probes, Carlsbad, CA), and measurement of superoxide production was done using DHE (Invitrogen, Molecular Probes, Carlsbad, CA), according to MEFs incubated under standard conditions with mitochondrial inhibitors (10 *μ*M), or a drug vehicle for 4 h. Cells were collected by trypsinization and washed twice with HBSS with 0.5% FBS. Cells were suspended in 1 ml of washing buffer and incubated with CM-H_2_ DCFDA (10 *µ*M) and DHE (10 *μ*M) for 30 min at 37°C. Fluorescence was measured immediately with FACSscan (Becton-Dickinson, Mountain View, CA). After data acquisition, CellQuest software (Becton Dickinson, Franklin Lakes, NJ) and WinList 5.0 (Topsham, ME) software were used to analyze ROS production.

### Mitochondrial DNA Content analysis

MtDNA content was measured in p53 WT (p53+/+) and disrupted p53 (p53-/-) PMEFs by SYBR green method (Invitrogen, Carlsbad, CA) in 7900HT Fast Real time PCR system (Applied Biosystems, Foster City, CA, USA). Standard curves were obtained using the normal mouse DNA and the reactions were performed in triplicates. Two sets of primers, one amplifying mtDNA and other amplifying nuclear DNA (Beta 2 microglobulin) were used. The ratio of the mtDNA compared to the nuclear DNA was used an index for measuring the mtDNA content.[31]

### Quantification of p53R2 levels

Real-time RT-PCR assays were performed using 7900HT Fast Real time PCR system (Applied Biosystems, Foster City, CA) with SYBR Green ER PCR Master Mix (Invitrogen, Carlsbad, CA). The primers for p53R2 were synthesized based on the published report[32] and PCR was performed according to the manufacturer's protocol in triplicates. The data was analyzed by RQ manager (Applied Biosystems, Foster City, CA, USA) software and the relative gene expression of the genes was made by comparing the results to the housekeeping gene (B2M) using the Delta delta Ct method.

### Immunoblotting analysis

Cells were lysed in RIPA lysis buffer: [50 mM tris pH 7.4, 150 mM NaCl, 1 mM PMSF, 1 mM EDTA, 1% triton × -100, 1% sodium deoxycholate and 0.1% SDS]. Immunoblot analysis of protein extracts was done as described elsewhere.[30] Cell-fractionation was done using a Pierce Mitochondrial Fraction Kit, according to the manufacturer's instructions (Pierce Biotechnology, Rockford, IL). Detection of p53 was done using mouse monoclonal antibody to p53 (p53(Ab-1) OP03 (Oncogene Research Products, Cambridge, MA) at a dilution of 1:1000. Ab-1 antibody recognizes an epitope (aa 371-380) at the C-terminus of p53. Detection of CoxII and laminin was done using mouse monoclonal anti-CoxII antibody (Invitrogen Molecular Probes, Carlsbad, CA) at a dilution of 1:1000 and anti-laminin A/C antibody (Santa Cruz Biotechnology, Santa Cruz, CA) at a dilution of 1:1000, respectively. The secondary antibody was peroxidase-labeled anti-mouse IgG (H+L) antibody (Vector Laboratories, Burlingame, CA). Anti-bovine a-tubulin antibody (Invitrogen, Molecular Probes, Carlsbad, CA) was used to assess equal protein loading.

## RESULTS

### p53 mediates mitochondrial control of the cell cycle

**Figure 1:**
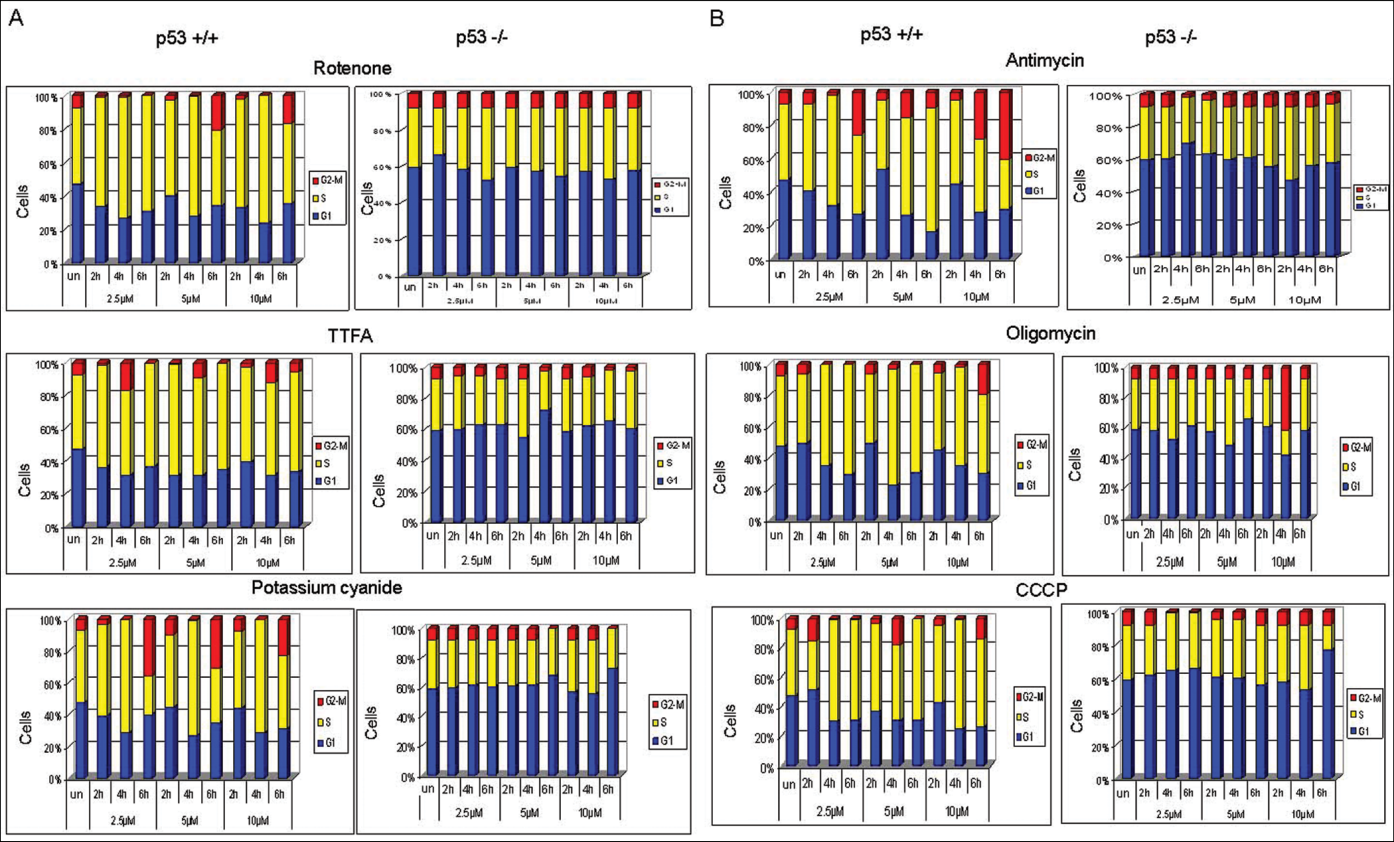
Effect of mito-I on cell-cycle distribution of primary MEF. A) Treatment with mito-I, rotenone and TTFA-induced S-phase delay in a time dependent manner. Potassium-cyanide treatment causes delay in the S-phase after longer than 2 h treatment. B) Treatment with mito-I, antimycin oligomycin and CCCP induced S-phase delay in a time-dependent manner. The primary MEFs derived from p53 null mouse embryos treated with mito-I did not exhibit significant changes in cell-cycle progression

We investigated whether mtOXPHOS regulates the cell cycle. We made use of a panel of inhibitors of mtOXPHOS function. Inhibitors were chosen based on their ability to inhibit specific steps of mtOXPHOS involving Complex I, III, IV or V. Using early-passage primary mouse embryo fibroblasts (primary MEFs) expressing wild-type p53, we investigated the dose- and time-dependent effect of mitochondrial inhibitors (mito-I) on the proliferative ability of the cells. Interestingly, exposure of MEF to mito-I induced S-phase delay in all cases. Among the mito-I used, rotenone and TTFA were the most effective, inducing delay in the S phase soon (two hours) after the treatment [Figure 1A]. The treatment of primary MEFs with other mito-I also delayed the S-phase, but after a longer exposure (six hours). The high dose of mito-I (10 *μ*M) caused an accumulation of cells in the G2/M phase of the cell cycle. As expected, the G2/M delay was accompanied by a decrease in the G1 and S phases of the cell cycle [Figure 1B].

The role of p53 in regulating mtOXHOS has already been demonstrated.[24] We therefore asked whether p53 mediates response to mitochondrial damage. We used p53 -/- primary MEFs derived from mid-gestation embryos.[33] Surprisingly, unlike wild-type, the primary MEFs derived from p53-/- mouse embryos did not exhibit significant delay in cell cycle progression after treatment with mito-I [Figures 1A and B]. These studies suggest a role for p53 in mitochondrial control of the cell cycle.

### ROS mediate p53-dependent cell-cycle delay

**Figure 2:**
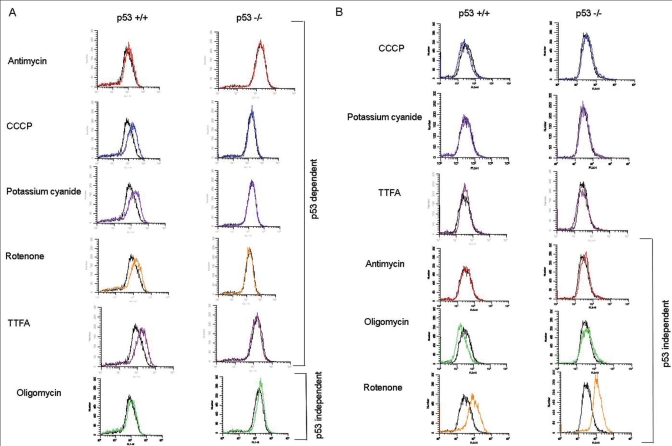
ROS production in primary MEFs treated with equimolar concentrations of mito-I. Cells were treated with equimolar concentrations of mito-I (10μM for 4h. A) p53-dependent production of H_2_O_2_ after treatment with antimycin, CCCP, potassium cyanide, rotenone and TTFA. Treatment with mito-I induced an increase in H_2_O_2_ production in primary MEF-expressing p53 but not in p53 null cells. B) No significant differences in O_2_^-^ production between p53 +/+ and p53 -/- pMEF cells treated with equimolar concentration of CCCP, potassium cyanide and TTFA were found. Lowe panel shows p53-independent production of O_2_^-^ after treatment with antimycin, oligomycin and rotenone

Mitochondria are the primary sites of reactive oxygen species.[34,35] In order to examine the source of cell cycle delay after mito-I exposure (10 *μ*M, for 4h), we measured H_2_O_2_ as well as superoxide (O_2_^-^) production in primary MEF cells. In all cases, treatment of p53 wild-type primary MEF with mito-I resulted in increased H_2_O_2_ production [Figure 2A]. In contrast, treatment of p53 -/- cells with mito-I (except for oligomycin) did not result in increased H_2_O_2_ production. Oligomycin treatment of isogenic p53 -/- primary MEF cells showed similar levels of increase in the H_2_O_2_ level as with p53 +/+ cells [Figure 2A]. We conclude: 1) mito-I treatment results in increased H_2_O_2_ production, and 2) that, except for oligomycin, mito-I induced H_2_O_2_, production is dependent on wild-type p53.

We also measured O_2_^-^ production in primary MEFs treated with mito-I. Interestingly, we did not observe significant differences in O_2_^-^ production between the p53 +/+ and p53 -/- pMEF when treated with CCCP, potassium cyanide and TTFA [Figure 2B]. However, the treatment of primary MEFs with other mito-I (antimycin, oligomycin and rotenone) led to a similar level of increases in O_2_^-^ production in p53 +/+ and p53 -/- cells. These studies suggest that increase in O_2_^-^ production led by inhibition of mtOXPHOS does not depend on the p53 protein. Together, the above studies suggest that 1) p53-dependent H_2_O_2_ production must play a key role in mitochondrial regulation of the cell cycle; 2) Since oligomycin produced similar levels of H_2_O_2_ and O_2_^-^ irrespective of p53 status, factors other than ROS may also play a role in mitochondrial control of the cell cycle.

### p53 expression after treatment with mito-I

**Figure 3:**
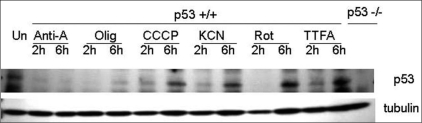
Mitochondrial damage modulates the level of p53 in primary MEFs. Western blot analysis of wild-type p53 cells treated with mito-I. Treatment initially leads to decreased expression of p53, but after six hours' exposure, the level of p53 becomes stabilized

The above studies suggest a role for p53 in mito-checkpoint response. Therefore, we determined the changes in p53 expression after mito-I exposure. Treatment of wild-type p53 cells with mito-I initially (after two hours) led to decreased expression of p53, but after six hours' exposure, the level of p53 became stabilized [Figure 3]. This pattern of p53 expression was observed with each mito-I used in the study. These studies suggest that the p53 expression plays an important role in response to the inhibition of mtOXPHOS.

### p53 translocation to mitochondria after treatment with mito-I

**Figure 4:**
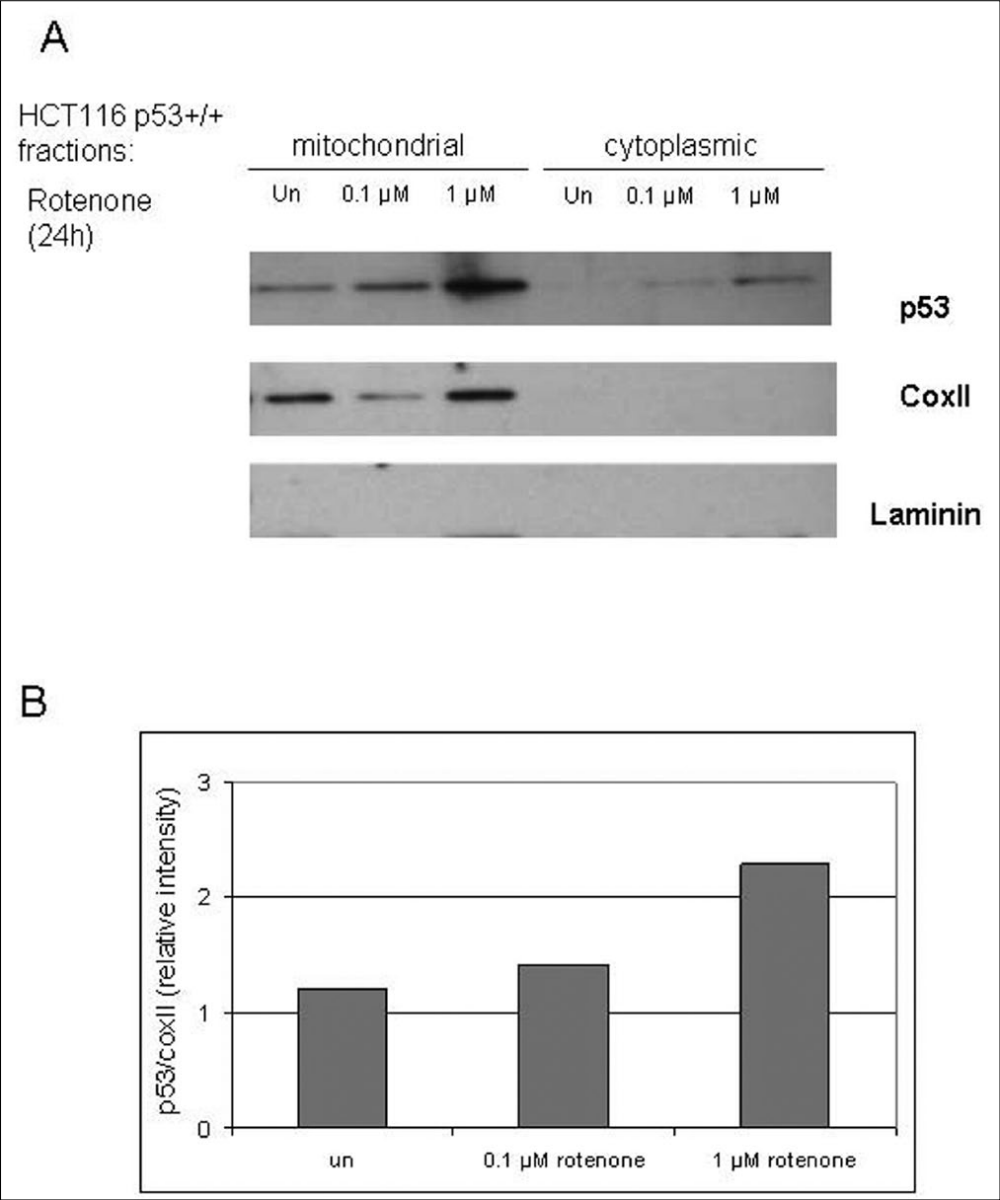
Mito-I inhibition leads to translocation of p53 into mitochondria. A) Treatment of cultured HCT116 p53 +/+ cells for 24 hours with rotenone results in a concentration-dependent increase in p53 protein levels in the mitochondrial fraction. B) Quantitation of the p53 signal in mitochondrial fraction induced by rotenone. The intensity of the p53 and CoxII bands was measured by densitometry

We evaluated the mitochondrial relevance of p53 in response to mito-I. We used rotenone, a complex I inhibitor of mitochondrial oxidative phosphorylation. The Western blot analysis shown in Figure 4A demonstrates that the treatment of cultured HCT116 p53 +/+ cells with rotenone results in a concentration-dependent increase in p53 protein levels in the mitochondrial fraction. Figure 4B shows the quantitation of the p53 signal by densitometry in mitochondrial fraction induced by rotenone. mtDNA encoded COXII protein was used to determine the quality of isolated mitochondrial fraction. Laminin was used to demonstrate lack of nuclear contamination in mitochondrial fractions. The treatment of cells with rotenone leads to a significant increase in ROS production (see above). These studies suggest that p53 plays a direct role in mito-checkpoint response.

### p53 regulates mtDNA copy number:

**Figure 5:**
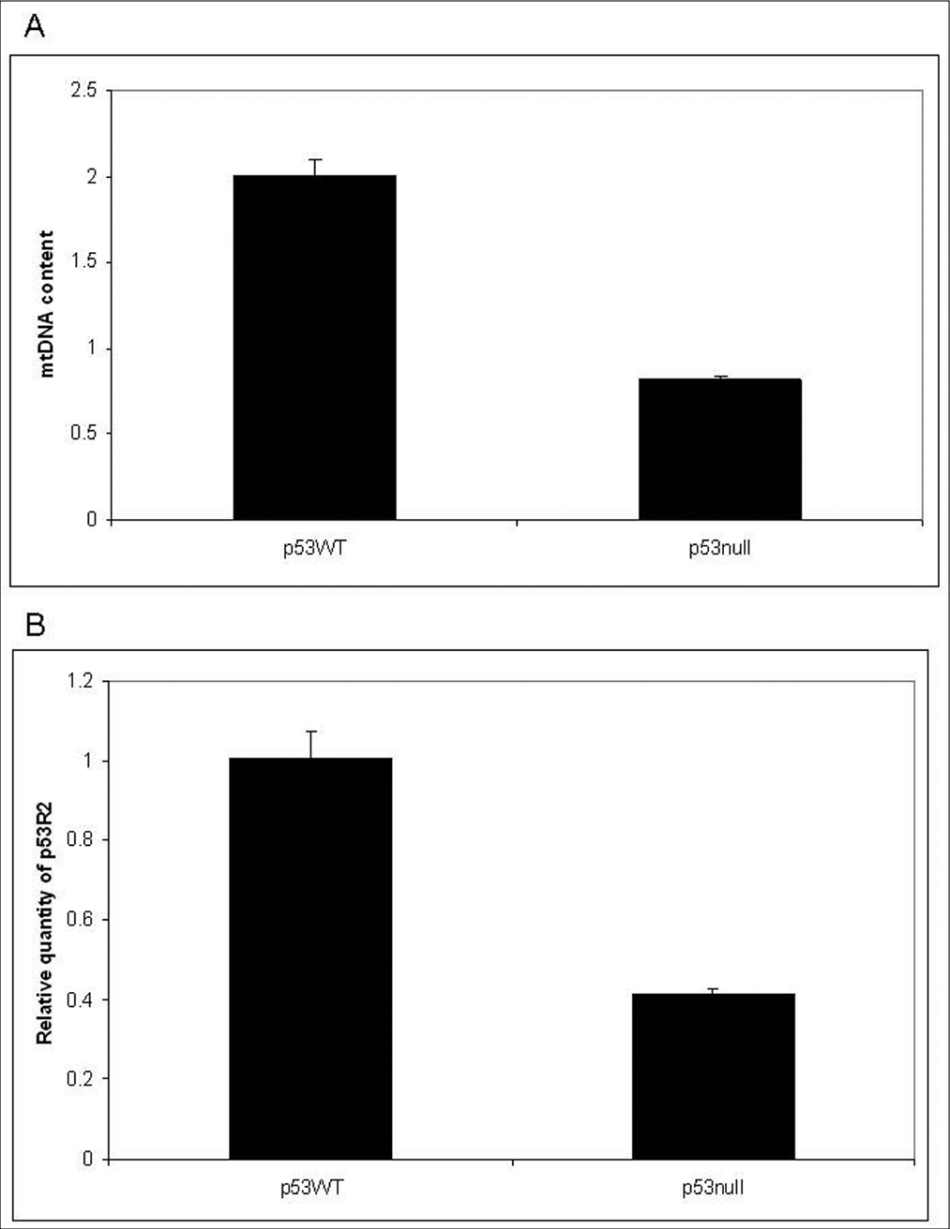
Decreased mitochondrial DNA content and downregulation of p53R2. A) Decrease in mtDNA content in p53-/- cells compared to p53 +/+ cells. B) Down regulation of the p53R2 in p53-/- cells compared to p53 +/+ cells

It has been suggested that p53 regulates OXPHOS activity. In particular OXPHOS activity was down regulated in p53-/- cells.[24] It has been clearly shown that the reduction in OXPHOS activity was not due to mutation in mtDNA.[24] A recent study suggested that mutations in p53R2, encoding a subunit of ribonuclease reductase (RR) cause mtDNA depletion syndrome in human.[36] We therefore asked whether p53 regulated mtDNA level in MEF cells. Figure 5A shows that p53 null contain approximately 50% less mtDNA copies when compared to p53 wild type cells. To identify the underlying reason for reduced mtDNA content we measured p53 regulated subunit of RNR, p53R2. Figure 5B shows a down regulation of the p53R2 which seems to contribute to the depletion of mtDNA. We conclude that loss of p53 leads to depletion of mtDNA in MEF cells.

## DISCUSSION

Mitochondria are essential cellular organelles. Damage to mitochondria resulting in dysfunction can have severe cellular consequences and can lead to human pathology. To ensure mitochondrial integrity, cells contain mitochondria-monitoring system(s) and response pathway(s) that either help avoid, repair or reverse damage to mitochondria. In order to unify these processes involved in protecting the integrity of mitochondria and, in turn, cellular integrity, the concept of a “mito-checkpoint” was recently proposed.[37,38]

Damage to mitochondria had been illustrated to result in epigenetic and genetic changes in the nuclear genome, resistance to cell-death and tumorigenic transformation.[10,11,16,39,40] Studies described in this paper suggest that disruption of the mitochondrial function in MEF cells induces S phase delay leading to G2/M phase arrest in the cell cycle. This observation is novel as many studies have reported a G1/S phase arrest in cell cycle in response to mtOXPHOS inhibition.[11,41–45] p53R2 encodes a small subunit 2 homolog of ribonucleotide reductase (RR). Transcription of other RR subunits such as R1 and R2 occurs exclusively during S-phase.[46] It is likely that altered expression of RR subunits contributes to S-phase delay observed in our study. p53 is known to play a key role in mediating cell cycle arrest after DNA damage.[47–49] It is unclear at this time whether the cell cycle response in this case is due to damage to DNA and that mtDNA is the primary target of damage. Our study revealed that ROS production increases after treatment with mito-I used in the study. Therefore, it is plausible that ROS-induced damage to mtDNA also plays an important role in this response.

We identified that, among the ROS, H_2_O_2_ is a key mediator because only H_2_O_2_ was increased after mito-I treatment in wild-type primary MEFs. Interestingly the levels of O_2_^-^ did not significantly change after treatment with CCCP, potassium cyanide and TTFA. These observations suggest that 1) mtOXPHOS inhibition leads to significant increases in H_2_O_2_ and 2) H_2_O_2_ is a major mediator in triggering mito-checkpoint-signaling in the cell. In this regard, it is worth noting an existing cross-talk between mitochondria and NADPH oxidase 1 (NOX1), a member of the family of NOX proteins involved in H_2_O_2_ production after the inhibition of mtOXPHOS.[30] To date, the NOX family of NADPH oxidases contains seven structurally related members that are homologous to the first NOX, named gp91 (now called NOX2). These family members include NOX 1 through 5 and DUOX1 and DUOX2.[30] These proteins play an intricate role in cellular signaling.[50]

Treatment of wild-type p53 MEF with mito-I resulted in the increased accumulation of ROS. This may be due to p53 regulation of critical enzymes involved in ROS metabolism.[51–53] A large group of pro-oxidant genes (which generate high levels of ROS) are regulated by p53. These genes include quinine oxidoreductases homolog *PIG3,* proline oxidase *PIG6* and ferredoxin reductase *FDRX,* whose products increase intracellular ROS. p53 also controls transcriptional regulation of antioxidant genes. These include p53R2.[54] Our study identified that p53R2 is down regulated in p53 -/- cells. Other antioxidant genes include microsomal glutathione transferase homologue *PIG12*, aldehyde dehydrogenase *ALDH4A*, glutathione peroxidase *GPX1*, Mn-superoxide dismutase *SOD2* and catalase. In addition, two members of the sestrin family, *SESN1* (PA26) and *SESN2* (Hi95), are also regulated by p53. Sestrins act as components of the peroxiredoxin regeneration system.[55] We do not yet know how p53-regulated target genes are affected by the inhibition of mtOXPHOS by mito-I. However, it is conceivable that an imbalance between the expression of pro-oxidant and antioxidant genes can contribute to production of ROS. Since mitochondrial OXPHOS activity is regulated by p53,[24,56] it is plausible that mitochondrial activity also contributes to ROS production and triggers the mito-checkpoint response. Taylor *et al.*[57] have reported that p53 is required to inhibit entry into mitosis when DNA synthesis is blocked. Since mitochondria are involved in nucleotide metabolism,[9,10] changes in the nucleotide pool can contribute to delay in DNA synthesis and, hence, delay in the S phase of the cell cycle observed in our study. Other mechanisms, such as mito-I-induced novel p53 gene targets, may also play a role. Increased transport of p53 to mitochondria in response to mito-I also further suggests a novel role for p53 as a mito-checkpoint protein. Consistent with these observations: 1) p53 regulates mtOXPHOS activity[24] 2) p53 regulates mitochondrial membrane potential[51] and biogenesis,[58] as well as 16S rRNA transcript encoded by mtDNA[59] 3) p53 enhances the accuracy of DNA synthesis in the mitochondria.[60] Studies described in this paper suggest that p53 also regulates mtDNA copy number.

Our study demonstrates that oligomycin, an ATPase inhibitor, produces similar levels of H_2_O_2_ and O_2_^-^, irrespective of p53 status. This suggests metabolic signals other than ROS may play a role in mitochondrial control of the cell cycle. Indeed, a decrease in ATP level is known to delay cell cycle progression in HL60 cells.[13] Interestingly, the levels of ATP reduction also determine the placement of cells in cell cycle arrest. A small reduction in ATP levels causes G1 arrest but increased reduction in the ATP level-leads to G2/M-phase arrest.[12,13] In *Drosophila,* Owusu-Ansah *et al*.[43] recently identified two signals involved in G1/S phase delay due to inhibition of the mitochondrial electron transport chain. One such signal involved an increase in AMP production and the other involved increased ROS.[42,43] It is unclear whether AMP status effects the p53 induced gene expression and brings about cell cycle arrest as observed in *Drosophila*. Additional studies are required to define the role of AMP in mitochondria induced cell cycle delay in mammalian cells.

**Figure 6:**
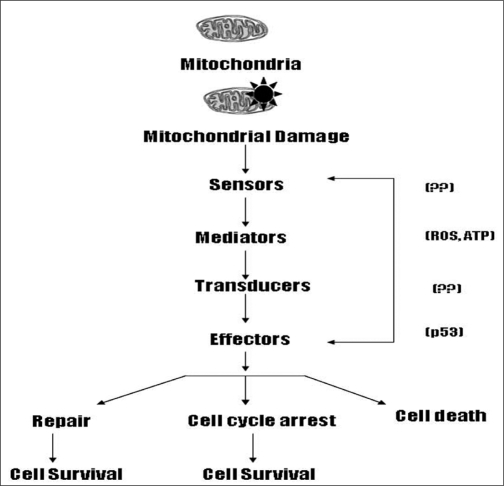
Conceptual organization of mito-checkpoint response. Mitochondrial damage is recognized by sensor protein(s). The signal is mediated by mediator(s) (such as ROS, ATP) and transduced by transducer(s). The transducers regulate effectors (such as p53). Together, mito-checkpoint helps to maintain the mitochondrial integrity of cell

Figure 6 describes how a mito-checkpoint can elicit cellular response to reverse or repair the damage to mitochondria. The mito-checkpoint can transiently block cell cycle progression while cells contain damaged/dysfunctional mitochondria. The mito-checkpoint may share characteristics of a signal-transduction pathway and may contain components, such as damage/dysfunction sensors, mediators, signal-transducers and effectors. Sensor protein(s) may recognize damage to mitochondria, and mediators signal the presence of damaged mitochondria and initiate a biochemical cascade(s). Transducers are likely to be protein kinases that can relay and amplify the signal. Effectors may include a transcription factor (such as p53 described in this paper), involved in regulation of cell cycle, DNA repair, and apoptosis.

## CONCLUSIONS

This paper describes that mitochondria regulate the cell cycle. However, it is unclear whether the reverse is true; i.e., whether the cell cycle regulates the mitochondrial cycle (i.e., fission, fusion, segregation and other aspects of mitochondrial biogenesis). Published literature provides evidence both in support of and against the integration of mitochondrial biogenesis with the cell cycle.[61] In light of the studies reported in this paper, it is important to revisit the question of integration of the mitochondrial cycle with the cell cycle. Given the large number of mitochondria in a single cell, it is possible that cells integrate the “mitochondrial cycle”[30] within the cell cycle, which are tightly regulated by genes involved in both cell cycle regulation and mitochondrial biogenesis, fission, fusion and segregation of mitochondria during various phases of the cell cycle. Studies are under way in our laboratory to identify these pathways involved in cell cycle regulation of mitochondrial biogenesis.

## AUTHOR'S PROFILE

**Dr. Singh** is one of the leaders in the field of mitochondria research and medicine. He is Professor of Oncology at Roswell Park Cancer Institute, the first cancer research, treatment and education center established in the United States. Prior to joining Roswell Park Cancer Institute, he was an Assistant Professor of Oncology and Environmental Health at Johns Hopkins School of Medicine. He is an expert in mitochondrial genetics and has more than 20 years of research experience. Dr. Singh is the author of over 65 research publications, including three books entitled Mitochondrial DNA Mutations in Aging, Disease and Cancer, Oxidative Stress, Disease and Cancer and Mitochondria and Cancer.

Dr Singh founded the Mitochondria Research Society, is the founding editor-in-chief of the journal Mitochondrion and is instrumental in promoting Mitochondrial Research and Medicine around the world. Dr. Singh has served or serves on the editorial boards of Cancer Biology & Therapy, Cancer Genomics and Proteomics, Technology in Cancer Research and Treatment, Nanomedicine, FEMS Yeast Research and Molecular Cancer and Journal of Oncology. He also serves as an expert peer reviewer for several other journals. He has served or serves as an expert panel grant of reviewers for U.S. Department of State, United Mitochondrial Disease Foundation, Komen Breast Cancer Foundation, and National Institutes of Health and other funding agencies such as National Research Council of Canada.
